# Creeping disaster along the U.S. coastline: Understanding exposure to sea level rise and hurricanes through historical development

**DOI:** 10.1371/journal.pone.0269741

**Published:** 2022-08-03

**Authors:** Anna E. Braswell, Stefan Leyk, Dylan S. Connor, Johannes H. Uhl

**Affiliations:** 1 School of Forest, Fisheries, and Geomatics Sciences, Institute of Food and Agricultural Sciences, University of Florida, Gainesville, Florida, United States of America; 2 Florida Sea Grant, University of Florida, Gainesville, Florida, United States of America; 3 Earth Lab, Cooperative Institute for Research in Environmental Sciences, University of Colorado Boulder, Boulder, Colorado, United States of America; 4 Department of Geography, University of Colorado Boulder, Boulder, Colorado, United States of America; 5 Institute of Behavioral Science, University of Colorado Boulder, Boulder, Colorado, United States of America; 6 School of Geographical Sciences and Urban Planning, Arizona State University, Tempe, Arizona, United States of America; Universidade de Aveiro, PORTUGAL

## Abstract

Current estimates of U.S. property at risk of coastal hazards and sea level rise (SLR) are staggering—evaluated at over a trillion U.S. dollars. Despite being enormous in the aggregate, potential losses due to SLR depend on mitigation, adaptation, and exposure and are highly uneven in their distribution across coastal cities. We provide the first analysis of how changes in exposure (*how* and *when*) have unfolded over more than a century of coastal urban development in the United States. We do so by leveraging new historical settlement layers from the Historical Settlement Data Compilation for the U.S. (HISDAC-US) to examine building patterns within and between the SLR zones of the conterminous United States since the early twentieth century. Our analysis reveals that SLR zones developed faster and continue to have higher structure density than non-coastal, urban, and inland areas. These patterns are particularly prominent in locations affected by hurricanes. However, density levels in historically less-developed coastal areas are now quickly converging on early settled SLR zones, many of which have reached building saturation. These “saturation effects” suggest that adaptation polices targeting existing buildings and developed areas are likely to grow in importance relative to the protection of previously undeveloped land.

## Introduction

### Increasing risk along coastlines

Coastal communities are increasingly threatened by sea level rise (SLR) and damaging flood events, growing problems resulting from climate change and the subsequent impacts from oceanic thermal expansion and melting of ice sheets[1]. Modelers project astounding future coastal risk in terms of damage, adaptation costs, migration, and population affected by flooding events [2–9]. Current estimates indicate that more than 2.5 million U.S. coastal properties, worth approximately $1.07 trillion dollars, are at risk of disruptive flooding by 2100 [10]. While these projected damage estimates are large enough to be comparable to the long-term aggregate growth of the US economy [11], they are also highly localized and result in some coastal areas being at much greater risk than others [12].

Reducing coastal risk to natural hazards requires advanced local adaptation strategies with respect to the built environment. Adaptation to sea level rise often revolves around local sustainable development and infrastructure strategies [13]. From the perspective of urban coastal planning, policy decisions can largely address two sets of issues: *how* communities build and *where* they build. Making effective decisions on these fronts requires a fact base that is rooted in the best available data on both hazards and the built environment [14]. These are particularly pressing considerations for coastal communities where urban inertia of prior building decisions [15] has important implications for whether communities adapt existing structures to hazards or focus on protecting shorelines from new development [16–21]. Until now the absence of temporally consistent, fine-scale data on historical building patterns has impeded such evaluations [19].

**Fig 1:**
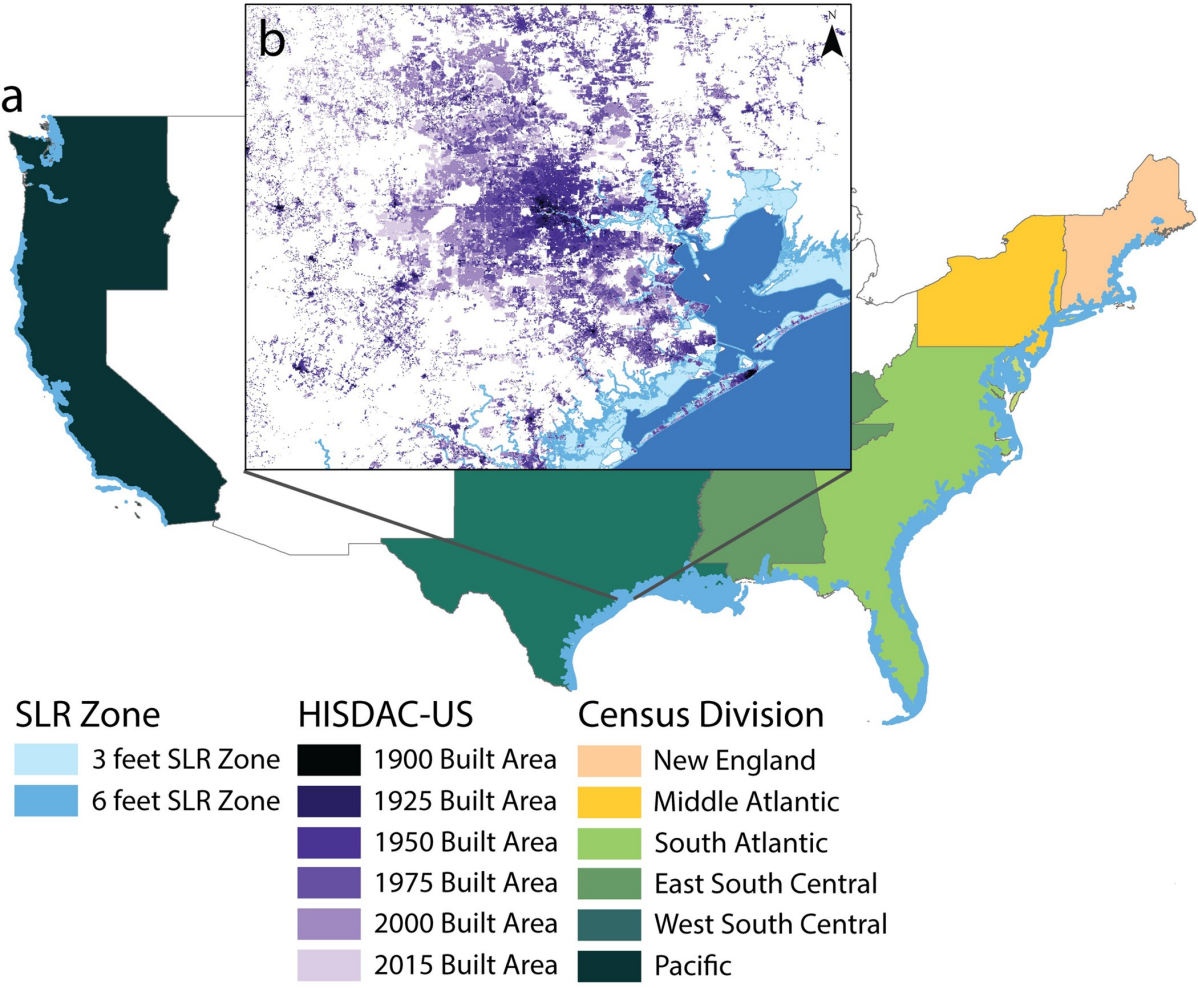
This analysis covers sea level rise zones (3 feet / 0.91 meters and 6 feet / 1.83 meters) across the conterminous United States [33] and by Census Division [34], with the 6 feet SLR zones displayed in blue (a). The spatial distribution of the year the first structure was built, provided by the HISDAC-US database [22], illustrates the historical development of cities, such as Houston, displayed in the inset (b). All data used in figure available through an open license for U.S. government datasets [33, 34] or through a CC0 license [22].

This paper leverages new multi-temporal data on building patterns, the Historical Settlement Data Compilation for the U.S. (HISDAC-US) [22, 23], across all major coastal communities in the United States from 1900 to 2015 (Fig 1). With this extensive and high resolution dataset, we ask how has the long-term development of coastal urban landscapes differed from non-coastal locations? And what role, if any, do coastal hazards play in shaping these differences? We address this question by undertaking a century-long analysis of urbanization encompassing both passive differences in regional building patterns between coastal and non-coastal places, and explicit responses to hazardous conditions (hurricane events). We first examine long-term changes in density and expansion across areas most likely to be impacted by coastal hazards, which we delineate using NOAA-defined SLR zones. Following this analysis, we explore how hurricane exposure relates to historical development and how current exposure levels are changing with recent building patterns. To the best of our knowledge, this is the first fine-scale nationwide analysis of the exposure of the coastal built environment to hazards over the twentieth century.

This work contributes to the separate but highly related fields of historical urban development and natural hazards. The natural hazards literature focuses on interactions between social drivers and environmental hazards that can lead to extreme events and drastically reshape social-environmental systems [24–26]. A growing body of social science is now focused on using big historical data to describe historical urban development patterns [27–29]. and linking these patterns to long-term social outcomes [30–32]. There is still little comparative work however in this historically focused, data-driven field that attempts to explain the conditions that give rise to different urban spatial forms, particularly with respect to environmental hazards. As we show below, we contribute to both fronts by establishing a robust link between coastal conditions, specifically hurricane activity, and the long-term spatial development of urban areas. Our analysis sets to stage for further ambitious long-term work that contends with the historical forces that have shaped our cities, and specifically incorporate the role of human-hazard interactions as a central feature of that research.

## Materials and methods

Using gridded historical settlement data layers derived from Zillow’s ZTRAX database [35], the Historical Settlement Data Compilation of the United States [22, 23], we analyze built-up property records across coastal regions of the U.S. from pre-1900 to 2015. Overlaying this data with sea level rise zones from the National Oceanic and Atmospheric Administration [33, 36], we investigate the trends of urban expansion into coastal areas at risk of the impacts of future sea level rise (Fig 1) and compare them with more inland areas across the conterminous Unites States, Census Divisions, and core-based statistical areas, including Census Micro- and Metropolitan Statistical Areas (MSA) [34]. We use measures including built-up property density, coastal land development (expansion) and the age of buildings, across sea level rise zones to investigate critical land transitions that inform the measurement of exposure of the built environment over the past century.

### Zillow’s ZTRAX dataset and historical settlement layers in HISDAC-US

Zillow’s ZTRAX database contains unique data on housing transactions, home values, rental estimates, spatial location, home- and property-related information (i.e., construction materials) as well as built-year information, for existing homes and certain other properties across the United States at the address level. As reported by the owner of the data, Zillow Group, Inc., the database includes information from more than 374 million public records and assessor data for approximately 200 million parcels in over 3,100 counties in the U.S.[28, 35]. The data are obtained from a major large third-party provider as well as through an internal initiative, called County Direct, which obtains records directly from the county assessor and recorders. These data provide unique opportunities for historical analysis of residential land use, housing markets and the built structure. Foremost of interest in this study, is information about residential, commercial and industrial land use related to built structures, building area (square footage), and the construction year. Furthermore, the ZTRAX database is a rich source of geospatial data in the form of approximate address point locations and address information enabling the characterization of settlement activity at fine spatial and temporal resolution. While the ZTRAX database is of unique nature covering most of the U.S., there are some limitations related to data quality, including spatial, temporal, and thematic uncertainties (see “Uncertainty and error in HISDAC-US” below).

Using the ZTRAX database, our team created new settlement data products covering the time period 1810–2010 (i.e., the HISDAC-US) and carried out an extensive uncertainty study [22, 37–41]. The data surfaces produced include a series of multi-temporal raster layers representing the built-up intensity (sum of gross indoor area of all built structures in a grid cell at a given year) [28], the year of the first settlement on record [37], the number of built property locations (BUPL) [39, 40], and accompanying (spatial, thematic, and temporal) uncertainty layers that were recently published as data products [42]. These gridded data layers were created using data integration methods at 250-meter spatial resolution and 5-year temporal resolution.

In order to better use the gridded datasets in combination with sea level rise zones, we resample the data to smaller grid cell sizes. Using the 250-meter original resolution, we resample the grid cells to a spatial resolution of 50 meters with a nearest neighbor technique. We then divide the number of built-up structures and built interior area raster values by 25 to reflect an equal portion of the original 250-meter grid cell in each target cell. We calculated the number of original (250 m) and resampled (50 m) grid cells within each SLR. On average, a SLR zone fits 1283 original grid cells (250 m), with 10% of SLR zones with less than 8 grid cells and 90% of SLR zones with less than 3,069 grid cells. SLR zones average 32,078 resampled grid cells (50 m), with 10% of SLR zones with less than 31 grid cells and 90% of SLR zones with less than 76,737 grid cells. While resampled cells do not represent the actual distribution of buildings within our highest resolution 250-meter grid cell, this up-sampling step allows us to better reflect the small, complex shapes of SLR zones without divulging more detailed proprietary data.

### Uncertainty and error in HISDAC-US

ZTRAX data is affected by issues of data incompleteness and generalization that is subsequently inherited by HISDAC-US [22]. The ZTRAX database can have two types of error: 1) commission errors where the database reports structures that do not exist, and 2) omission errors where structures exist that are not reported in the database [22, 41]. For example, Morgan City, Louisiana has no records of structures and built year (the year when the structure was built) available; these data are necessary for mapping development through time. Previous work shows that in urban areas and after 1850, the datasets show high levels of reliability when compared to a variety of validation datasets, including building footprint data from Microsoft and historical US census housing counts [41]. In this study, we exclude states and Metropolitan Statistical Areas (MSAs) that had significant area proportions missing (more than 50%; see S1 Fig in S1 File). In addition, we use only the best quality data at the grid cell level for all regional and state analyses (grid cell-level, year built missingness < 5%) to ensure the processes and change patterns measured are reliable and accurate. We assessed missingness by using the TPixMiss data layer in HISDAC–US [42]. TPixMiss contains the number of records with missing year built for each cell value through time to understand temporal completeness [22]. We also assume that year built reflects the first building and not the replacement of old buildings. In prior work, we demonstrated that this survivorship bias may be problematic for neighborhood-scale analysis, but these issues are far less consequential for more aggregate analysis, such as the urban scale analysis presented here [23, 41, 42]. There is an exception in this study where changes in built environment compositions represent a consequence of post-hurricane rebuild activities (see” Hurricane impacted areas are denser and more developed than unaffected areas” section below). As demonstrated below, we can overcome this limitation and even use it as an advantage in the analyses comparing trajectories of built structure and age across different places (affected and unaffected by extreme events).

Relying on the best quality data available in the ZTRAX database (>95% data completeness) precludes all coastal counties in Louisiana and restricts other counties (see S1 Fig in S1 File). The low data quality grid cells account for approximately 14.5% of the area within coastal MSAs and 17.2% of the built-up properties recorded in ZTRAX in coastal MSAs. Even with excluding Louisiana and a few counties in Florida and South Carolina, we still find massive growth in the Southeast over recent decades with this more conservative approach. Therefore, we have more confidence in the findings. The variability in the data from raw ZTRAX to resampled and cleaned 50-meter grid cells ranges depending on the quality of data at the location (S1 Table in S1 File). While the presented estimates are conservative and a subset compared to other studies [10], this strategy allows us to establish and focus on highly reliable assessments of changes and trends in the built environment at local and regional scales.

### NOAA sea level rise zones

In order to track the development of areas at risk of coastal hazards, we use SLR zones as areas at high risk of hazards over this century. These areas are close to coastal waterbodies, and therefore more likely to be impacted by coastal storms and hurricanes. We use the SLR zone instead of FEMA flood zones because of the equal treatment across the country. FEMA flood zones are often out of date and created with different methods across the country based on funding, population and natural hazard probability [43]. For our purposes, we employ the NOAA Sea Level Rise dataset [33] to determine the boundaries of the 3 feet and 6 feet zones which come as state-level datasets. We use two zones in order to understand the differences between areas that are highly likely to be affected (3 ft/0.91 m) and areas that could be affected in the worst climate scenarios (6 ft/1.83 m). There are some limitations to the NOAA dataset, as it is created using a modified bathtub approach [36], which assumes all areas will be equally affected by SLR (equal rise in a bathtub) without accounting for variations in geomorphology, shoreline change and other factors. The associated drawbacks are less important for our analysis which uses the zones to understand large-scale development trends.

### Metropolitan and micropolitan statistical areas

To aggregate our results to representative areas along the coast, we use Metro- and Micropolitan Statistical Areas (MSAs) [34], which allow us to determine boundaries of contiguous population cores within consistent 2010 boundaries for all years. For this analysis, we employ MSA boundaries to analyze settlement patterns in SLR zones. Metropolitan statistical areas are defined as adjacent counties or county equivalents that have one or more urban areas with a population of at least 50,000 and any adjacent areas that have a high degree of integration with the urban area, measured by commuting [44]. Micropolitan Statistical Areas are similarly defined except they have an urban core area with a population greater than 10,000, but not more than 50,000. Although much of the area considered in the MSA boundaries in 2010 was rural in 1900, we use contemporary MSA boundaries to assess how these transitional units developed over time, similar to recent approaches of urban change assessments [45, 46]. We use MSAs as the unit of analysis because most of the coastline is covered by Metropolitan and Micropolitan Statistical Areas (S1 Fig in S1 File).

### HURDAT2 database

We use NOAA’s National Hurricane Center’s North Atlantic hurricane database (HURDAT2) [47] to determine which MSAs were affected by hurricanes over our study period. We employ the records in the dataset from 1900 to 2015 with hurricanes that made landfall along the Gulf and Atlantic coasts of the United States. To determine which MSAs were affected by hurricanes, we intersect the MSAs with all hurricane eye landfalls, and select any coastal MSA that was within 20 km of an eye’s landfall. Because of the uncertainty in the location of landfall in the HURDAT2 database (e.g. landfall location offshore), we determine the 20 km buffer was adequate to identify MSAs close to or containing hurricane eye landfall locations and thus a direct impact from the storm event. We also count the number of hurricane hits and their severity for each MSA from 1900 to 2015. These data capture most major hurricanes since 1900. These hurricanes can be an early indicator (through storm surge) of future SLR conditions. Therefore, we use them in this paper to understand development of areas that are frequently exposed to flooding and damage in comparison to other areas.

### Constraining variables

To account for areas of the SLR zones that would not likely be developed, we exclude some areas from our analysis. In order to determine which lands are protected, we use the Protected Areas Database for the United States [48]. We also use the National Wetlands Inventory [49] to exclude areas that are considered wetland, and less likely to be developed. These areas are erased from the SLR zone before we calculate development metrics.

For each SLR zone and MSA polygon, zonal statistics are used to determine the total number of structures, built interior area (indoor square meters of buildings within the grid cell) and built grid cells (grid cells with at least one property record) for each polygon in five year increments from 1900 to 2015. Zonal statistics are computed for each state’s SLR zone, the SLR zone within each MSA and the remaining area of the MSA outside of the SLR zone (inland MSA). These data layers are used to derive all variables and carry out all analysis for MSAs, SLR zones, states and the entire U.S. coastline over time.

### Derived variables

**Fig 2:**
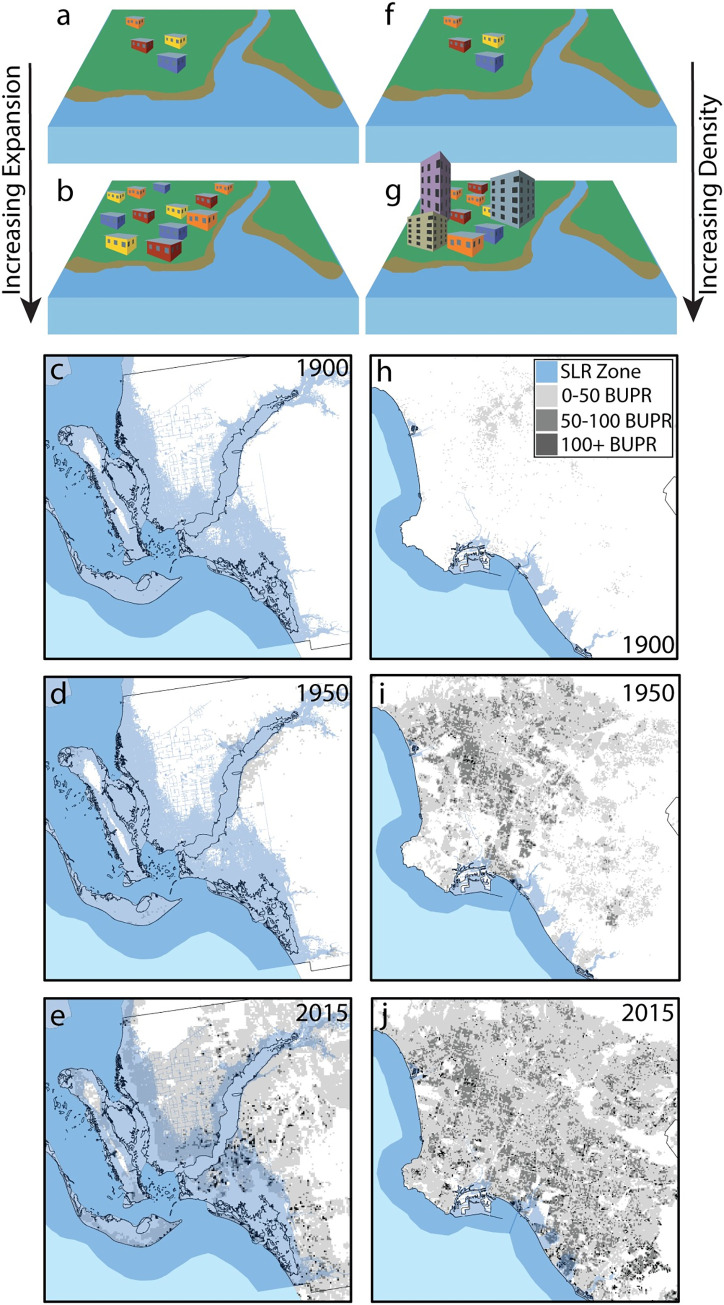
The two major drivers of urban development, measured in this paper are: 1) expansion (panel a to b), or the aerial growth of developed land, and 2) structure density (panel f to g), or the addition of more or bigger structures to already developed areas. An example of high expansion within SLR zones [30] is in Fort Meyers, FL where developed land rapidly expanded after 1950 (panels c-e). Los Angeles, CA is an example of high growth of structure density within SLR zones, particularly after 1950 (panels h-j). Both examples are measured using the built up properties (BUPL) data layer from HISDAC [39]. All data used in figure available through an open license for U.S. government datasets [33, 34] or through a CC0 license[39].

We create several variables from HISDAC-US to compare measures of development. We aggregate all data by state within the entire conterminous United States and by MSA within each census division from 1900 to 2015. To create a trajectory of built area, we sum built grid cells and divide them by the total number of grid cells in the unit of analysis. This measure provides the proportion of grid cells developed, binned by 5-year windows (Built Area Proportion (*BAP*) = *A*_*p*_*/A*_*T*_; where *A*_*p*_ is the number of built grid cells and *A*_*T*_ is the total number of grid cells in the unit of analysis). To determine density of structures within developed land found in the unit of analysis, we divide the number of structures by the total number of developed grid cells for each 5-year time window (Structure Density = *S/A*_*p*_; where *S* is the number of structures and *A*_*p*_ is the total number of developed grid cells in the unit of analysis; Fig 2).

To understand expansion and land cover change across communities, we measure shifts in the built-up proportion of areas over time. Expansion is a measure of the increase of the proportion of built grid cells during a given time period (Expansion = *BAP*_*t2*_*—BAP*_*t1*_; Fig 2). To derive a relative measure of added density in relation to expansion, we develop a densification measure that is a ratio of the number of added structures to the number of added developed grid cells:

$$\frac{S_{t2}-S_{t1}}{A_{p\;t2}-A_{p\;t1}}$$

where *S* is the number of structures and *A*_*p*_ is the number of developed grid cells in the unit of analysis. When this measure is greater than one, more structures are being added than grid cells; when densification is less than one more developed grid cells are being added than structures.

## Results

### Development trends across the coastal United States

We begin our analysis by describing the continental building trends along U.S. coastlines. Across the conterminous United States, developed area and housing stock dramatically increased along the coastline over the past century. Based on the highest-quality data in our settlement layers, we find that much of this growth occurred between 1950 and 2000 with a 446% increase in structures and a 220% increase in developed land area. Much of this trend reflects the high rates of suburban expansion and new growth in Sunbelt states after the Second World War [28, 50]. This highest-quality data subset contains approximately 1,050,000 built structures of any type (commercial, residential, etc.), and 35% of the land in the 6 foot SLR zone is developed (with at least one structure per grid cell) across the conterminous U.S in the year 2015. The median year of construction of those buildings in the 6 feet SLR zone is 1975.

**Fig 3:**
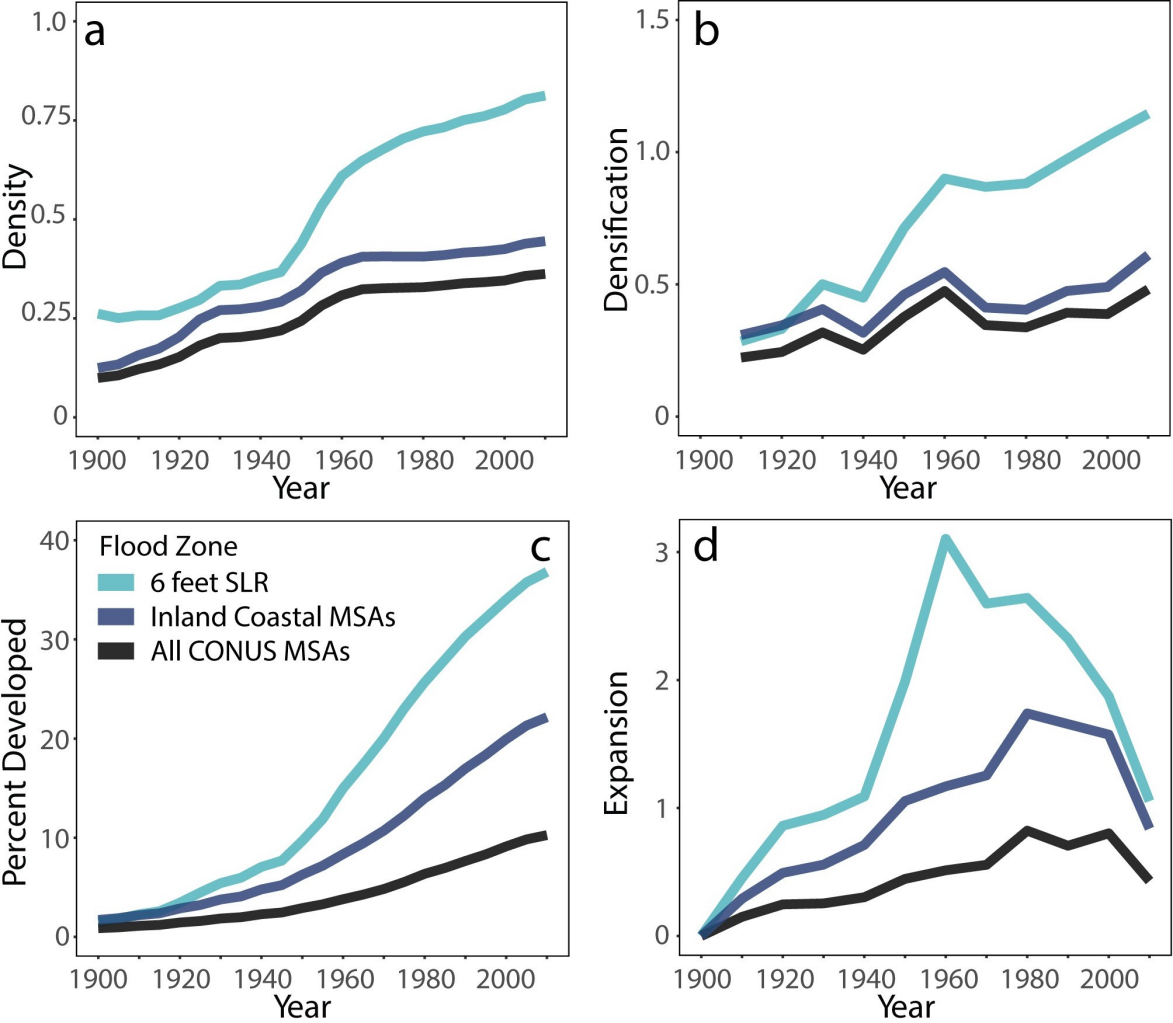
The 6 feet SLR zone, the inland part of coastal Micro- and Metropolitan Statistical Areas (MSAs; without SLR zone), and all MSAs of the conterminous United States (CONUS) show over time: a) increasing density (structures per grid cell) of developed area; b) recent increases in densification (structures added relative to expansion; values greater than 1 mean that density is increasing faster than the addition of developed land grid cells; c) a steady increase in built-up percentage (percentage of grid cells developed within the MSA or SLR zone); and d) changing rates of expansion of developed area (increase in developed grid cells) with steep increases between 1950 and 1970, and dramatic decreases after 1980. Densification (b) and expansion (d) were calculated over 10-year time increments. Compared to all MSAs across CONUS, SLR zones and coastal MSAs are denser, more developed, and consistently have higher rates of expansion than MSAs across the country as a whole.

In the mid-twentieth century, coastal building transitioned from high rates of expansion to higher levels of densification, as urban areas grew into available space and added more structures to already developed areas [23, 29]. SLR zones across the United States have higher structure density, are more developed, and have densified and expanded at higher rates than inland coastal areas and MSAs across the country (Fig 3). Density, or the number of structures per grid cell (250m resolution), increased dramatically in SLR zones after 1950 (Fig 3A), with a slowing, but still positive trend after 1980. Mirroring trends in density, developed land in coastal communities greatly expanded after 1950, slowing significantly after 1980 (Fig 3C). Expansion of coastal communities (measured by the proportion of land developed per time period) created SLR zones that are, on average (mean), 35% developed in the 6-feet zone (Fig 3C and 3D). After the high expansion of the first part of the 20^th^ century, expansion rates dropped after around 1980. Rates of added structures were higher than the development of new land later in the study period, with densification (number of structures added per developed grid cell during the time period of interest) greater than one after 1980 (Fig 3B).

**Fig 4:**
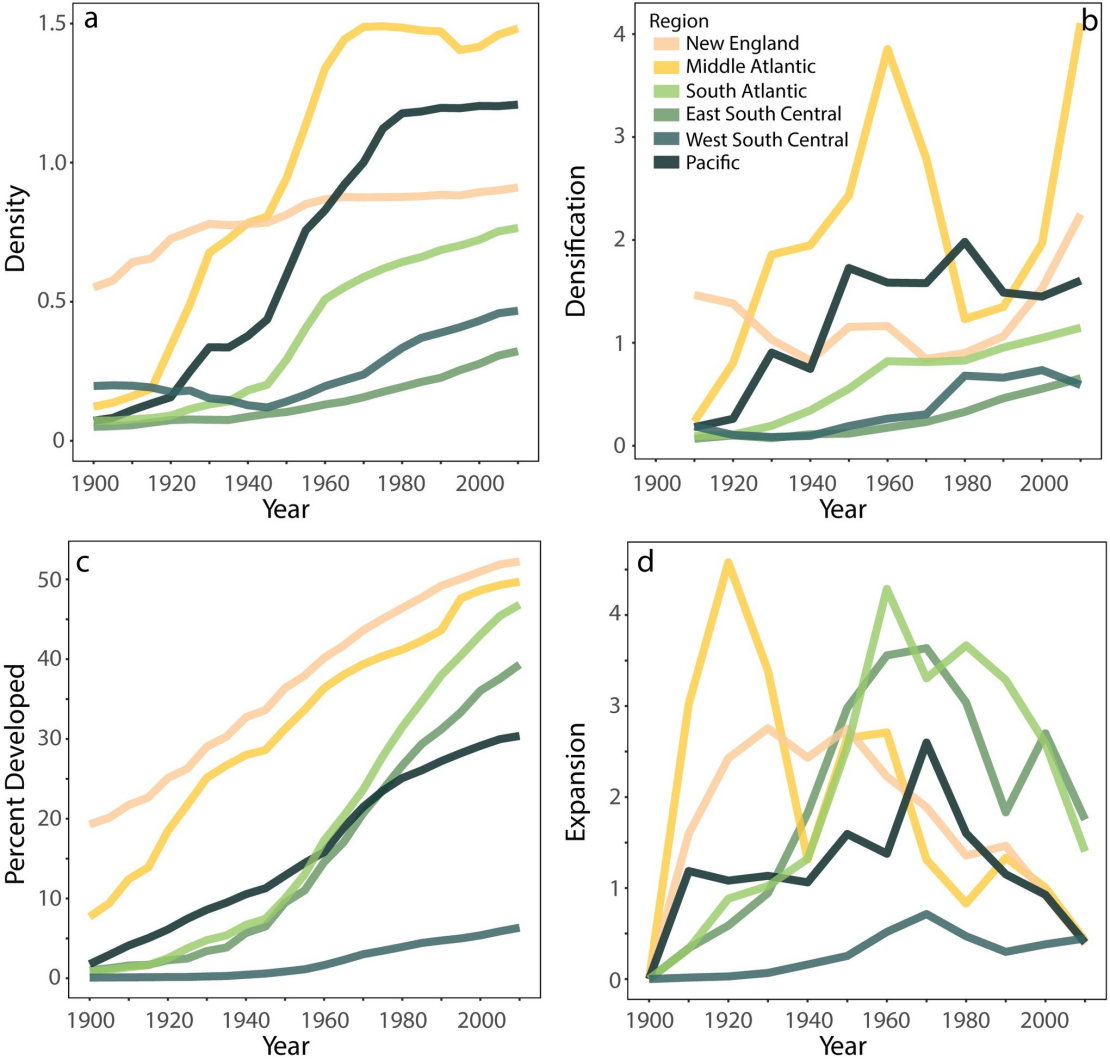
**Regional trends in the 6 feet SLR zone tell a varied story about the timing and intensity of historical coastal development.** Built environment characteristics are based on structure density (structures per developed grid cell); a); densification (structures added relative to expansion; values greater than 1 mean that density is increasing faster than the addition of developed land grid cells); b); proportion of the SLR zone developed (proportion of grid cells developed within the unit of measurement); c); and expansion (increase in developed grid cells); d). Colors denote coastal MSAs aggregated to Census Divisions. Densification (b) and expansion (d) were calculated over 10-year time increments.

Although coastlines across the country differ from inland areas, coastal development also varies by region. When we consider patterns of expansion and densification by census division, some nuanced regional trends appear (Fig 4). Older coastal areas, such as the Northeastern region of the U.S. (New England and the Middle Atlantic Divisions), developed early, even preceding our study period, and rank high in density and developed area throughout the 20th century. The coasts of the Southeastern (South Atlantic and East South Central Divisions) and the Pacific U.S. had high rates of growth after 1950, mirroring sunbelt growth trends [51], closing the gap and converging to the already developed Northeast in built environment characteristics. Despite increases in density and percent built, the West South Central remains lower in density of structures and percent of SLR zone developed.

Among those regions, we find different types of development. The Pacific division overtook New England in structure density within developed land yet remains low in percent of SLR zone developed. The Southeast has an opposite growth pattern with high expansion later in the 20^th^ century and SLR zones that are increasingly developed. These two growth patterns illustrate the distinction between densification- and sprawl-based development trajectories. The Western Gulf coastline (West South Central Division) also developed rapidly after 1950, but remains less developed than the Northeast, Pacific and Southeast. The relatively modest historical intensity of development in the Western Gulf makes it a potential hotspot for new building and land-use transitions in the future.

### Changing patterns of coastal built environment exposure

**Fig 5:**
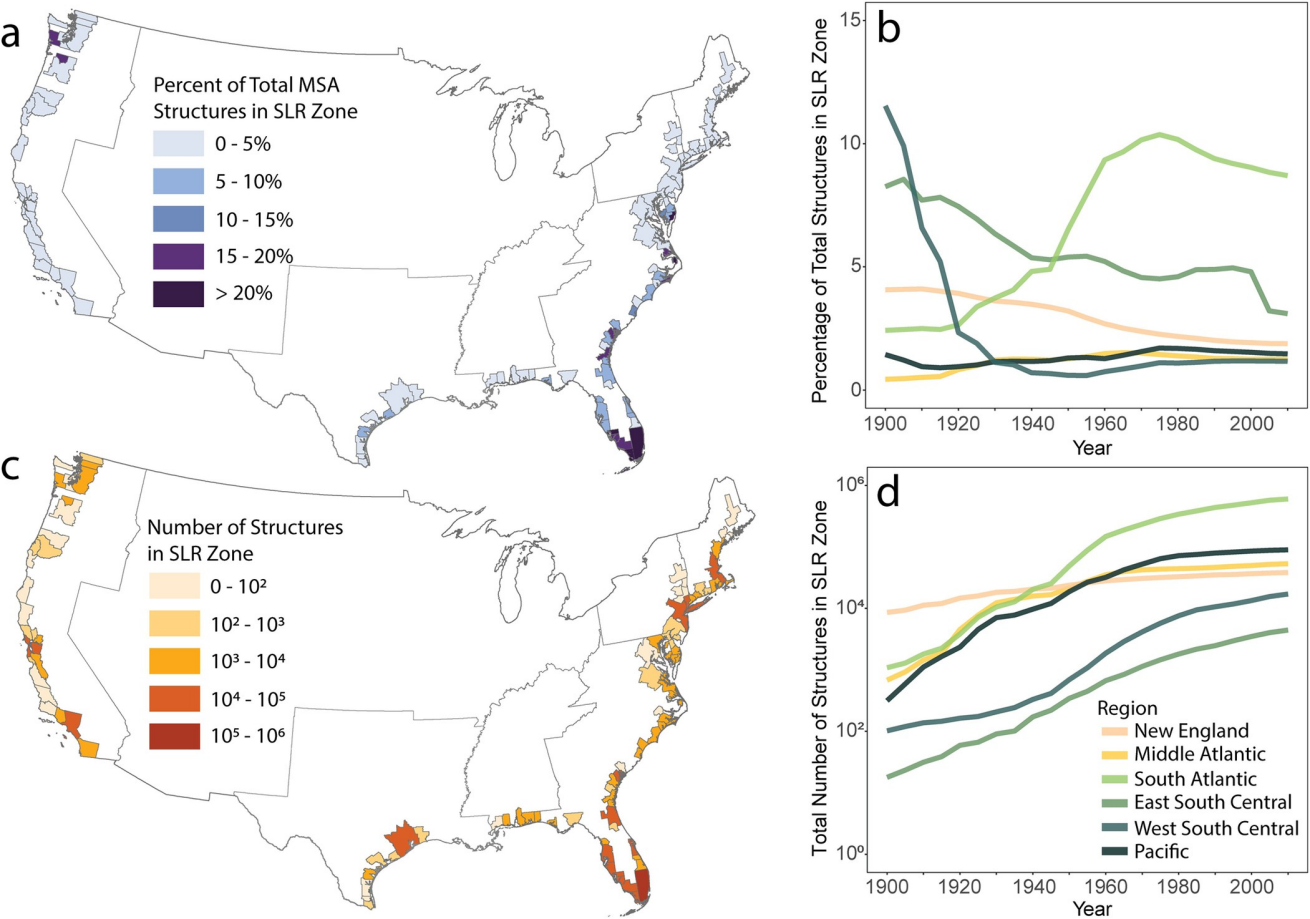
The number of structures in the 6 feet SLR zone is used as an indicator of built environment exposure, illustrating high exposure, particularly in Florida and the South Atlantic division. Relative built environment exposure (the percentage of total structures in the MSA contained in SLR zone) is depicted in MSAs in 2015 (a) and over time by Census Division (b). Absolute built environment exposure (absolute number of structures in SLR zone) is illustrated by MSA in 2015 (c) and over time by Census Division (d). All data used in figure available through an open license for U.S. government datasets [34] or through a CC0 license [39].

In the previous section, we showed that the coastlines–particularly those in the 6-foot SLR zone–are more developed than inland areas. The addition of buildings in these places has slowed over recent decades. How is this shifting trend affecting exposure to environmental change? We answer this question by quantifying the absolute and relative exposure of MSAs to SLR, by comparing the total number of buildings (absolute exposure; Fig 5C and 5D) to the percentage of buildings (relative exposure; Fig 5A and 5B) inside SLR zones.

Relative exposure decreased or remained relatively constant across the study period in most divisions, except for the South Atlantic. Absolute exposure increased across the entire study period, slowing between 1970 and 1980, with the South Atlantic surpassing both the Northeast and Pacific in total number of structures in the SLR zone. While relative exposure remained steady or began to decrease, the absolute number of structures in SLR zones increased across all divisions.

We find that the South Atlantic division has the highest percentage of total MSA structures in the SLR zone (Fig 5B), with areas like Miami, FL and Hilton Head Island, SC driving this trend (Fig 5A). Since about 1970, the percentage of total MSA structures in the SLR has dropped in the South Atlantic because of increasing addition of structures in inland areas. These observations provide evidence for the development of coastal areas first and the subsequent expansion into more inland areas in the same MSAs. Additionally, we find that areas at highest risk of hurricanes, the South Atlantic, have higher exposure across both measures, showing that hurricane affected areas may be more developed than unaffected areas.

### Hurricane impacted areas are denser and more developed than unaffected areas

We now investigate the likelihood that the unique environmental conditions of coastlines have influenced local building patterns. We address this question by rigorously testing how one coastal natural hazard, hurricanes, affects local development. We assess if the building trajectories in SLR zones of urban areas differ from their respective interior areas, based on their environmental context. This comparison between the SLR and interior zones of the *same* urban areas helps account for historical and geographic differences in building that may otherwise be unrelated to the environmental questions of concern here.

**Fig 6:**
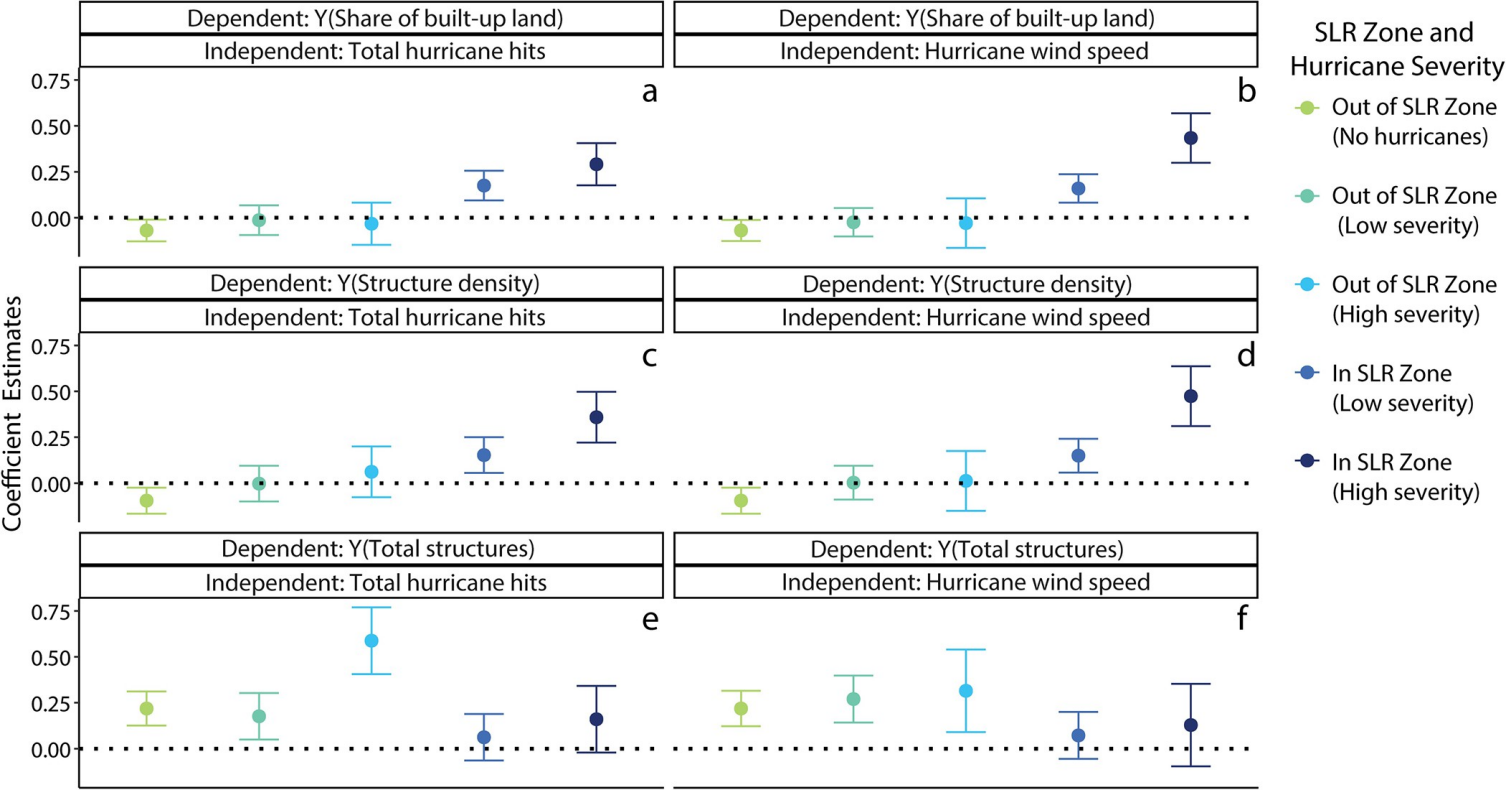
Results from six regressions (a-e) using 3 dependent variables (share of built-up land, structure density, total structures) x 2 independent variables (wind speed, total historical hits) across five units of analysis (MSA zones within and out of the SLR and no, low, or high impact from hurricanes). The dependent variables were measured in 2015 and the models include a census division fixed effect. The coefficient estimates (y-axis) from the regressions are all relative to a SLR zone with no storms in the database (the zero line in the graphs). In models that use wind speed, MSAs classified as low severity were hit by less severe storms that have average max wind speeds < 100 mph; high severity MSAs were hit by severe storms, those that have average max wind speeds > 100 mph (b, d, f). In the regression models using hurricane hits (a, c, e); severe levels of storm hits are defined as places hit by 5 or more storms since 1900, less severe levels are those with 1–4 storm hits since 1900. Higher coefficient estimates illustrate higher rates of structure density or total structures relative to MSAs not affected by hurricanes.

We first provide a descriptive illustration of the building-hurricane interactions that we are attempting to explain. Using a regression, we present estimates predicting 2015 building attributes across places characterized along two dimensions: 1) whether or not these locations are in the 6-foot SLR zone, and 2) the historical severity of the recorded storms, as measured by cumulative hurricane count and average windspeed (Fig 6). Differences in building patterns by these two severity measures are consistent: SLR zones historically exposed to more intense cyclonic activity tend to have higher proportions of built-up land and higher structure density (Fig 6). We find no notable difference in total structure count across storm severity and SLR zones (Fig 6). From a descriptive perspective, we find that hurricane activity tends to be associated with more compact and more dense building patterns. This descriptive analysis is limited, however, in determining whether these distinct building patterns reflect adaptation to cyclonic activity or a potentially wide range of omitted factors, such as the region or development time period. To better identify the impact of hurricanes on the built environment, we examine the timing of local building activity with hurricane events. We do this through a within-metropolitan analysis of historical building patterns and hurricane trends.

We construct this analysis by dividing coastal MSAs in two parts: the area inside and the area outside of the 6-foot SLR zone. We then stack these locations and their ZTRAX attributes based on 5-year time steps from 1900 to 2015 (24 observations per location within a metropolitan area). Using these data, we estimate a series of two-way fixed effect panel regression models [52] of the following form:

$$y_{it}=\alpha +\beta_{1}{(SLRZ}_{i}x\;{Storm}_{it})+\beta_{2}{(SLRZ}_{i})+\beta_{3}({Storm}_{it})+X_{i}\Gamma +\varepsilon_{it}$$

where the outcome variables *y* for coastal areas *i* include one of three measures of building intensity: total structures; share of built-up land; and density of structures. The main effect of *SLRZ_i_* estimates differences between metropolitan areas in and out of SLR zones. We expect that *β*_2_ > 0 if SLR zones are more intensively built-up than non-SLR zone areas. The main effect *Storm_it_* is a measure of storm severity based on the count of storm landfalls recorded in the HURDAT2 database from 1900 to 2015 [47]. Due to the consistency of our estimates in terms of hurricane events and mean wind speed (Fig 6), here we focus only on cumulative hurricane events (Table 1). Our coefficient of interest is *β*_1_, which tests whether SLR zones experienced higher growth in building intensity following hurricanes. Our intuition is that if hurricane-prone environments develop distinctive building patterns, these differences will be identifiable within MSAs, between the SLR zone and the remainder of the metro area.

**Table 1:** Built environment and cumulative storm hits panel regression analysis from 1900–2015.

Location	Coastal		Coastal &	Coastal		Coastal &	Coastal		Coastal &
			> 0 landfalls			> 0 landfalls			> 0 landfalls
	(1)	(2)	(3)	(4)	(5)	(6)	(7)	(8)	(9)
	Share of	Share of	Share of	Structure	Structure	Structure	Total	Total	Total
	built-up land	built-up land	built-up land	density	density	density	structures	structures	structures
Sea-level rise zone	0.0775*	0.0507*	0.0428*	0.0572*	0.0375**	0.0102	-84.43*	-62.86*	16.68
	(0.012)	(0.013)	(0.023)	(0.012)	(0.014)	(0.018)	(20.347)	(21.011)	(24.783)
Cumulative storms	0.0232*	0.00786	-0.00422	0.0341*	0.0228*	0.000326	34.14*	46.50	46.42*
	(0.006)	(0.006)	(0.008)	(0.010)	(0.008)	(0.007)	(18.045)	(28.164)	(23.441)
SLRZ x Cumulative storms		0.0307*	0.0327**		0.0226**	0.0295**		-24.71	-44.98*
		(0.010)	(0.013)		(0.010)	(0.012)		(21.141)	(24.731)
Constant	0.111*	0.124**	0.0931	0.223*	0.233*	0.164*	284.1*	273.3*	141.0*
	(0.057)	(0.057)	(0.056)	(0.051)	(0.054)	(0.036)	(94.408)	(86.197)	(45.687)
Observations	4656	4656	2016	4656	4656	2016	4656	4656	2016
*R* ^2^	0.705	0.726	0.766	0.558	0.571	0.711	0.405	0.416	0.554
Clustered SEs	MSA	MSA	MSA	MSA	MSA	MSA	MSA	MSA	MSA
Fixed effects	Year,	Year,	Year,	Year,	Year,	Year,	Year,	Year,	Year,
	Year x CD,	Year x CD,	Year x CD,	Year x CD,	Year x CD,	Year x CD,	Year x CD,	Year x CD,	Year x CD,
	MSA	MSA	MSA	MSA	MSA	MSA	MSA	MSA	MSA

Standard errors in parentheses.

* *p* < 0.10, ** *p* < 0.05, * *p* < 0.01.

Notes: Columns 1, 2, 4, 5, 7 and 8 are estimated from the full sample of MSAs with coastal areas inside the 6-foot SLR zone. Columns 3, 6 and 9 focus only on MSAs with at least one hurricane landfall in our data. Standard errors are clustered at MSA scale and fixed effects include individual year, MSA and individual year x census division. Thus, the variation in the interaction term primarily comes from within-MSA differences over time. See S2 Table (S1 File) for even more conservative but supportive estimates.

We control for geographical and temporal heterogeneity between metropolitan areas by using the vector *X_i_*. These controls include individual year and MSA fixed effects, and time-varying building trends within census divisions. These controls adjust our estimates for general and regional time trends, with the identifying variation primarily stemming from differences within MSAs over time. If hurricane activity leads to differences in building between SLR zones and interior areas, we expect *β*_1_> 0. To provide further confidence in our estimates, we also include results from when the sample is restricted to MSAs with at least one hurricane landfall in our data.

We find that MSAs affected by hurricanes are denser and more developed than those not affected by hurricanes (Table 1; Fig 6). As indicated by the estimates, we find that cumulative hurricane activity is associated with significantly higher rates of building in terms of the proportion built area (Table 1, Col. 2) and structure density (Table 1, Col. 4). In terms of the share of built-up land, the SLR zone of MSAs tend to be around 8 percentage points more built up than their respective interior areas (Table 1, Col. 1). Every one-unit increase in the accumulation of storms over time is associated with an increase of 2.3 percentage points in share of built up land. The positive interaction term and the attenuation of the main storm effect toward zero indicates that the impact of storms on the built-up share of land is largely concentrated in the SLR zones of MSAs (Table 1, Col. 2). These estimates closely resemble those based on structure density (Table 1, Col. 5) and are consistent with the estimates generated from the more restrictive samples (Table 1, Col. 3; 6).

We find a negative but less precise effect of hurricane activity on structure count (Table 1, Col. 7–9). Although our estimates show fewer structures in SLR zones and more structures in storm-prone areas, the within-metropolitan difference estimate is negative but not statistically significant. It is notable, however, that this interaction term is significant and larger in magnitude in the more restrictive sample, implying that storm activity is associated with fewer built structures among hurricane-hit locations. Taken together with our findings on density and proportion built, our estimates indicate that hurricane-prone environments may produce more dense, compact urban landscapes. This finding also identifies the need for further studies at the local-scale to confirm this national-scale observation.

## Discussion

### Development along U.S. coastlines leads to increasingly dense coastal zones

Our settlement trajectories reveal that vast amounts of the United States coastline were developed during the economic boom after WWII (Fig 3) [50]. With much of the U.S. population, large urban centers, and industry located along the coast, our findings support the claims of others that population and building footprints will continue to expand along the coastline [3, 53, 54]. How exactly these development patterns will play out remains to be seen.

Development across the coastlines of the United States has varied temporally and geographically. Our analysis reveals two different ways *how* coastal settlements typically develop: expansion (the lateral growth of developed areas) and densification (the addition of structures to already developed areas). Across time, we show that coastal building tends to expand with low-density construction in the early phase of development but has transitioned to densification from roughly 1980 on. In the modern era, the U.S. coastlines added structures and increased in density.

All coastal regions follow this general trend, albeit staggered through time with differences in the onset of expansion and densification. This trend is particularly apparent in New England, where expansion is high through 1960, at which point it declines as densification increases (exceeding one), indicating that structures are added to developed land units at a higher rate than new land developed after 1980 (Fig 4). No regions currently show increasing expansion, pointing to growing densification (addition of structures) and resulting in increasing built environment exposure along the coast. These observations also might reflect a possible exhaustion of developable land in the SLR zone in our study areas. In addition, this saturation effect may be exacerbated by a possible increasing resistance to new land development, driving increasing densification of already developed areas.

Regardless of the cause of contemporary growing densification along coastlines, this trend leads to two relevant outcomes: 1) Increasing exposure through added structures and increased square footage within SLR zones; and 2) It implies possibly decreasing built environment vulnerability through less land expansion into hazardous areas and more modern building codes. The implications of this trend are that if these newer structures are built with more restrictive building codes than older structures or protected under federal flood insurance, then they could be more adaptive to coastal hazards than older structures [16, 18, 19]. SLR zones were developed more intensely and faster than other urban areas around the country, continuously growing the exposure to natural hazards in these zones.

In assessing *where* our coastlines developed, our findings indicate increasing convergence of the density and expansion characteristics of SLR zones across regions [55]. This convergence creates coastal communities with similar exposure, a finding that shows the dense development of the United States coastlines regardless of regional likelihood of hazard. Convergence patterns imply that the effects of the historical conditions that gave rise to distinctive coastal built environments may have been temporary. We anticipate increasing uniformity in exposure conditions over the years to come. The growth patterns within the Sunbelt over the last century (including the Southern Pacific, East South Central, West South Central and South Atlantic Divisions) lead to divisions with development characteristics that became increasingly similar to the Northeast. The Southeastern coastline is almost at 50% built-up proportion in the SLR zone, and the Pacific even exceeds the coastal density in New England (Fig 4). The advent of air conditioning, military base proliferation, and increasing commonness of automobile and air travel further drove the development of the Sunbelt with its attractive environmental amenities [51, 55–58]. With increasing expansion and density, in the coastal communities of the Sunbelt, these trends suggest convergence in the profiles of more recently developed regions with the older Northeast.

### Coastal built environment exposure is highest in areas at elevated risk of natural disasters due to SLR

The number of structures in the SLR zone is a general indicator of the physical exposure of a community to impacts from coastal storms and flooding [59]. For example, metropolitan areas such as Miami, New York, Boston, Houston, Los Angeles, and San Francisco contain high numbers of structures and high structure density in SLR zones and are thus highly exposed to coastal hazards. The South Atlantic and Florida continue to add structures at high rates and have a larger relative exposure than other divisions, despite being at high risk of natural hazards (i.e., hurricanes; Fig 5D) [24, 60]. This result is particularly critical given that there is a high probability of hurricanes in southern Florida [60, 61] and high susceptibility to flooding due to low elevation [62]. Built environment expansion and densification corresponds with previous results about population movement to the Sunbelt and into hurricane prone areas [51, 63]. With increasing numbers of structures and property at risk in SLR zones, absolute exposure of structures to natural hazards and flooding is increasing across hazardous coastlines.

### Post-hurricane development is associated with increased built environment exposure to coastal hazards

The Sunbelt region rapidly developed after WWII. We find that areas within the Sunbelt are growing and becoming denser despite cumulative hurricane hits and high risk of natural hazards. Recent evidence points to the construction of larger structures after hurricane disasters along the East and Gulf Coasts [54]. Our results complement this finding, as we identify increasingly dense SLR zones in areas affected by hurricanes in recent decades, despite the widespread recognition of coastal hazards. In short, areas at risk of hurricane impacts are more highly developed than those that are not at risk.

Our findings imply that U.S. coastlines in 2015 look different if they have been hit by even one hurricane. These coastal communities look even more different if they accumulate more than one hurricane hit during the study period. Just as multiple hurricanes affect community processes, such as likelihood of hurricane evacuation [64] or population growth [65], we find that these disasters also affect the physical structure of the built environment in hurricane-prone areas. This development effect could be the outcome of rebuilding and adaptation after impacted, older buildings are damaged or of the impetus for communities to build back after natural hazard [66]. Other studies find that building footprints increase after hurricane events, increasing development intensity and building sizes along the coast [56]. In Florida, residential exposure increased and development did not slow down after coastal development plans and restrictions were enacted [67], pointing to variations in the effectiveness of building restrictions along disaster-prone areas. This finding is relevant for future planning as not only are coastal hazards associated with increases in exposure, but the densified nature of this development will also constrain future adaptation efforts with respect to climate change.

### Knowledge of exposed built structures and their characteristics enables new forms of risk assessment of the built environment

**Fig 7:**
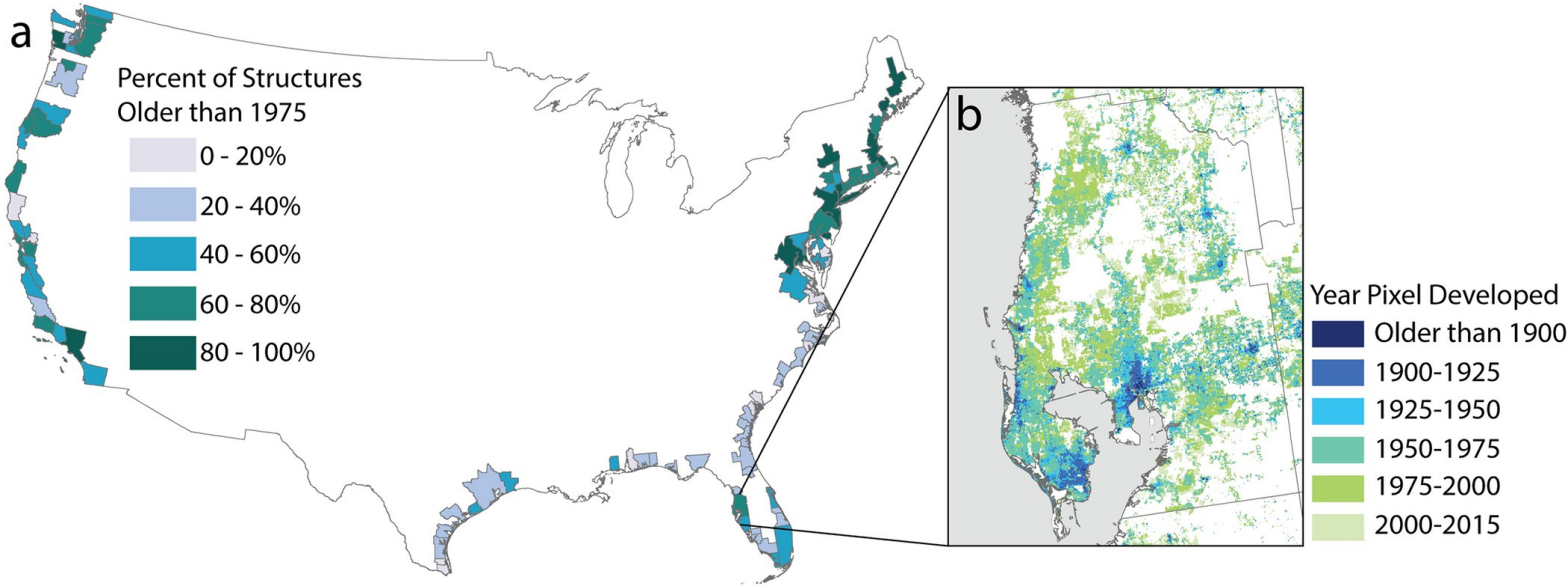
The year a building is constructed can give an indication of the building codes and practices of the time, and thus, built environment vulnerability. Here we show the percent of structures older than 1975 across MSAs (a). We illustrate the fine scale detail provided by HISDAC-US [22] to understand the development and age of neighborhoods in Tampa, FL (b). All data used in figure available through an open license for U.S. government datasets [34] or through a CC0 license [37].

Guided by an interest to identify measures that contribute to the exposure and vulnerability of the built environment, we highlight the use of additional characteristics of built structures, such as age, in hurricane-affected areas (Fig 7). The presence of old structures is a possible indicator of elevated vulnerability due to the lack of adaptation in building codes and old construction practices [19, 68–70]. Older coastal areas, particularly in the Northeast, were developed in time periods when housing codes and building techniques were not as advanced or adaptive as today [18, 19]. This basic metric of building age has the potential to help identify metropolitan areas along the U.S. coast or even neighborhoods within MSAs that are vulnerable (i.e., highly susceptible to damage) to the impact of natural hazards along the coast (Fig 7). It also shows that density and development patterns do not illustrate the complete story of exposure. Building condition and adaptation also drives built environment vulnerability, characteristics we are unable to assess with current datasets.

Considering age data in fine detail (Fig 7B), we can identify neighborhoods and grid cells in cities like Tampa that are old and possibly built with less adaptive capacity to SLR. Using HISDAC-US data layers, we can determine built environment characteristics at very fine spatial and temporal resolution [22] and use a multitude of attributes about the built environment [28]. Locations, timing of construction and composition of the built environment are all important factors to assess the vulnerability of those communities. Admittedly, such fine-resolution analysis requires particular care due to the issues of data completeness described above. Nonetheless, developing new metrics from these layers has the potential to expand our understanding of the vulnerability and exposure of the built environment at highly localized scales.

Our findings point to the need for policies and incentives that prioritize adaptation in our coastal communities. The coastlines of the United States are densely developed and saturated. In some places, accommodation of rising water levels through structural alterations, such as piers or sea walls, may be enough for reduction of risk [71]. Nature-based approaches are also an option for areas where marshes or mangroves are alternatives to grey infrastructure [72]. In areas where coastal hazards and exposure are very high, retreat from the coastline may be the only adaptation choice available [71, 73]. All these alternatives rely on capital to create coastal communities adapted to sea level rise and hazards. Our results imply that investment in adaptation along our already saturated coastlines is necessary for future exposure and risk reduction.

## Conclusions

Exposure of coastal communities has led to staggering losses and damages from natural hazards [18, 19, 69], yet these coastal areas continue to grow in size and number of structures. We show that coastal communities have denser and more intensely developed built environments than more inland areas, leading to high exposure, regardless of probability of natural hazards. These findings have specific implications for the implementation of adaptive building codes and coastal development policies. The rate of exposure growth has slowed across all divisions, possibly due to saturation of coastal areas and decreasing availability of developable land. Our findings imply that protection of developable land, particularly in places that have not experienced expansion (i.e., the Gulf Coast), is critical to minimize exposure across our coastlines. In addition, these results reinforce the crucial need for adaptation in already saturated and dense coastal communities.

While these changes could point to a possible policy opportunity to focus on building codes for new buildings to improve adaptation of coastal areas [74], for many regions the damage is already done. Planning in major coastal cities will need to focus on retrofitting and protecting existing structures in densely developed areas. Locations with older houses, such as the coastlines of the Northeast, would particularly benefit from adaptive measures and community infrastructure to decrease built environment vulnerability. Moreover, similar to other studies, our analysis shows that building size and density has tended to increase after hurricanes hit an area [54], meaning that coastal exposure to hazards followed compounding trajectories. It is therefore imperative to avoid “business as usual” coastal development scenarios and use this new evidence to support more sustainable development, building policies, and coastal retreat after disaster.

Other regions, particularly along the Gulf Coast (i.e., West and East South Central Divisions), have low density and small proportions of built-up land. Such areas still have much scope to ring-fence coastal development through building policies and codes that restrict development of hazardous areas such as SLR zones. These actions would reduce the growth of exposure to coastal hazards and the costs of protecting or adapting buildings in the future. Future research should also consider how socio-economic status can affect exposure and vulnerability to hazards in combination with development trends. We know that structures alone do not predict damage outcomes after hazards and understanding the interactions between the built environment and social system is critical for determining risk.

Our research points to saturation along many parts of our coastline, where expansion has decreased and structure addition remained steady, thus resulting in high-density built environments. Geographic differences in coastlines do play a role in shaping the built environments of coastal communities. We are creating an environment where the number of people and the cost of natural disasters continue to grow within constrained areas where biophysical exposure is increasing. The United States’ aging infrastructure, older buildings, and increasing density can lead to extremely exposed and vulnerable coastlines in the future. Through capitalizing on the spatial and temporal resolution of this new settlement data compilation, we begin to understand the evolution of exposure along the coast, *how* communities build and *where* they build, and how this knowledge can be used for planning ahead for more sustainable and resilient coastal communities.

## Supporting information

S1 File The supplemental information file contains the following tables and figure. The S1 Table is an analysis of built up properties by a subset of counties. We used the ZTRAX raw data, 250 meter grid cells from HISDAC-US (BUPL; Uhl and Leyk 2020), and resampled grid cells used in the analysis for this paper to compare the number of structures across datasets. The S2 Table is the results from the built environment and cumulative storm hits regression analysis from 1900–2015 (with MSA-Year fixed effects). S1 Fig shows the Metro- and Micro- Statistical Areas (MSA) included in analysis. ZTRAX data is affected by issues of data incompleteness. We exclude states and MSAs from this study that has a significant percentage of grid cells (more than 50%) with low quality data (grid cell year built missingness < 5%). All data used in figure available through an open license for U.S. government datasets [30].
(DOCX)

## References

[1] IPCC. Climate Change 2013: The Physical Science Basis. Contribution of Working Group I to the Fifth Assessment Report of the Intergovernmental Panel on Climate Change. Stocker TF, Qin D, Plattner G-K, Tignor M, Allen SK, Boschung J, et al., editors. Cambridge, United Kingdom and New York, NY, USA: Cambridge University Press; 2013. doi: 10.1017/CBO9781107415324

[2] Hallegatte S, Green C, Nicholls RJ, Corfee-Morlot J. Future flood losses in major coastal cities. Nature Climate Change. 2013;3: 802–806. doi: 10.1038/nclimate1979

[3] Hauer ME, Evans JM, Mishra DR. Millions projected to be at risk from sea-level rise in the continental United States. Nature Climate Change. 2016;6: 691–695. doi: 10.1038/nclimate2961

[4] Hinkel J, Lincke D, Vafeidis AT, Perrette M, Nicholls RJ, Tol RSJ, et al. Coastal flood damage and adaptation costs under 21st century sea-level rise. Proceedings of the National Academy of Sciences. 2014;111: 3292–3297. doi: 10.1073/pnas.1222469111 24596428 PMC3948227

[5] Robinson C, Dilkina B, Moreno-Cruz J. Modeling migration patterns in the USA under sea level rise. PLoS ONE. 2020;15: 1–15. doi: 10.1371/journal.pone.0227436 31968017 PMC6975524

[6] Buchanan MK, Kulp S, Cushing L, Morello-Frosch R, Nedwick T, Strauss B. Sea level rise and coastal flooding threaten affordable housing. Environmental Research Letters. 2020;15: 124020. doi: 10.1088/1748-9326/abb266

[7] Kulp S, Strauss BH. Rapid escalation of coastal flood exposure in US municipalities from sea level rise. Climatic Change. 2017;142: 477–489. doi: 10.1007/s10584-017-1963-7

[8] Balk D, McGranahan G, Anderson B. Urbanization and ecosystems: Current patterns and future implications. The New Global Frontier. London: Routledge; 2012. pp. 197–216.

[9] Pesaresi M, Ehrlich D, Kemper T, Siragusa A, Florczyk A, Freire S, et al. Atlas of the human planet 2017: Global exposure to natural hazards. 2017 [cited 30 Jun 2021]. Available: http://repo.floodalliance.net/jspui/handle/44111/3706

[10] Union of Concerned Scientists. Underwater: Rising Seas, Chronic Floods, and the Implications for US Coastal Real Estate. Cambridge, MA; 2018. Available: https://www.ucsusa.org/sites/default/files/attach/2018/06/underwater-analysis-full-report.pdf

[11] Dinan T. Projected Increases in Hurricane Damage in the United States: The Role of Climate Change and Coastal Development. Ecological Economics. 2017;138: 186–198. doi: 10.1016/j.ecolecon.2017.03.034

[12] Climate Central. Ocean at the Door: New Homes and the Rising Sea, 2019 Edition. 2019. Available: https://ccentralassets.s3.amazonaws.com/pdfs/2019Zillow_report.pdf

[13] Laukkonen J, Blanco PK, Lenhart J, Keiner M, Cavric B. Combining climate change adaptation and mitigation measures at the local level. Habitat International. 2009;33: 287–292.

[14] Meerow S, Woodruff SC. Seven Principles of Strong Climate Change Planning. Journal of the American Planning Association. 2020;86: 39–46. doi: 10.1080/01944363.2019.1652108

[15] Lorie M, Neumann JE, Sarofim MC, Jones R, Horton RM, Kopp RE, et al. Modeling coastal flood risk and adaptation response under future climate conditions. Climate Risk Management. 2020;29. doi: 10.1016/j.crm.2020.100233 32832376 PMC7433032

[16] Dehring CA, Halek M. Coastal building codes and hurricane damage. Land Economics. 2013;89: 597–613.

[17] Fronstin P, Holtmann AG. The determinants of residential property damage caused by Hurricane Andrew. Southern Economic Journal. 2019;61: 387–397.

[18] FEMA. Hurricanes Irma and Maria in the U.S. Virgin Islands: Building Performance Observations, Recommendations, and Technical Guidance; FEMA P-2021. 2018. Available: https://www.fema.gov/media-library/assets/documents/170486

[19] FEMA. Mitigation Assessment Team Report: Hurricane Sandy in New Jersey and New York, FEMA P-942. 2013. Available: https://www.fema.gov/media-library/assets/documents/85922#

[20] Armstrong SB, Lazarus ED. Reconstructing patterns of coastal risk in space and time along the US Atlantic coast, 1970–2016. Natural Hazards and Earth System Sciences. 2019;19: 2497–2511. doi: 10.5194/nhess-19-2497-2019

[21] Solecki W, Grimm N, Marcotullio P, Boone C, Bruns A, Lobo J, et al. Extreme events and climate adaptation‐mitigation linkages: Understanding low‐carbon transitions in the era of global urbanization. WIREs Climate Change. 2019;10: e616. doi: 10.1002/wcc.616

[22] Leyk S, Uhl JH. HISDAC-US, Historical settlement data compilation for the conterminous United States over 200 years. Scientific Data. 2018;5: 1–14. doi: 10.1038/sdata.2018.175 30179234 PMC6122163

[23] Uhl JH, Leyk S, McShane C, Braswell A, Connor D, Balk D. Fine-grained, spatio-temporal datasets measuring 200 years of land development in the United States. Earth System Science Data. 2021;13: 119–156. doi: 10.5194/essd-13-119-2021 34970355 PMC8716019

[24] Iglesias V, Braswell AE, Rossi MW, Joseph MB, McShane C, Cattau M, et al. Risky development: Increasing exposure to natural hazards in the United States. Earth’s Future. 2021; e2020EF001795. doi: 10.1029/2020EF001795 34435071 PMC8365714

[25] Balch JK, Iglesias V, Braswell AE, Rossi MW, Joseph MB, Mahood AL, et al. Social-Environmental Extremes: Rethinking Extraordinary Events as Outcomes of Interacting Biophysical and Social Systems. Earth’s Future. John Wiley and Sons Inc; 2020. doi: 10.1029/2019EF001319

[26] McPhillips LE, Chang H, Chester M v., Depietri Y, Friedman E, Grimm NB, et al. Defining Extreme Events: A Cross-Disciplinary Review. Earth’s Future. 2018;6: 441–455. doi: 10.1002/2017EF000686

[27] Markley SN, Holloway SR, Hafley TJ, Hauer ME. Housing unit and urbanization estimates for the continental U.S. in consistent tract boundaries, 1940–2019. Scientific Data. 2022;9: 82. doi: 10.1038/s41597-022-01184-x 35277512 PMC8917187

[28] Leyk S, Uhl JH, Connor DS, Braswell AE, Mietkiewicz N, Balch JK, et al. Two centuries of settlement and urban development in the United States. Science Advances. 2020;6: eaba2937. doi: 10.1126/sciadv.aba2937 32537503 PMC7269677

[29] Uhl JH, Connor DS, Leyk S, Braswell AE. A century of decoupling size and structure of urban spaces in the United States. Communications Earth & Environment. 2021;2. doi: 10.1038/s43247-020-00082-7 34970647 PMC8716013

[30] Connor DS, Storper M. The changing geography of social mobility in the United States. PNAS. 2010;117: 30309–30317. doi: 10.1073/pnas.2010222117/-/DCSupplemental PMC7720141 33199616

[31] Leonard B, Smith SM. Individualistic culture increases economic mobility in the United States. Proceedings of the National Academy of Sciences. 2021;118. doi: 10.1073/pnas.2107273118 34493672 PMC8449392

[32] Boustan LP, Kahn ME, Rhode PW, Yanguas ML. The effect of natural disasters on economic activity in US counties: A century of data. Journal of Urban Economics. 2020;118. doi: 10.1016/j.jue.2020.103257

[33] NOAA. Sea Level Rise Data. 2017. Available: https://coast.noaa.gov/slrdata/

[34] Census Bureau U.S. TIGER/Line Shapefiles—MSA. 2010. Available: https://www2.census.gov/geo/tiger/TIGER2010/

[35] Zillow. ZTRAX: Zillow Transaction and Assessor Dataset, 2017-Q4. In: Zillow Group, Inc. [Internet]. 2017. Available: http://www.zillow.com/ztrax/

[36] Noaa. Detailed Methodology for Mapping Sea Level Rise Inundation. 2012; 5. Available: http://www.csc.noaa.gov/slr/viewer/assets/pdfs/Inundation_Methods.pdf

[37] Leyk S, Uhl JH. Historical settlement composite layer for the U.S. 1810–2015. In: Harvard Dataverse [Internet]. 2018. 10.7910/DVN/PKJ90M

[38] Leyk S, Uhl JH. Historical built-up intensity layer series for the U.S. 1810–2015. In: Harvard Dataverse [Internet]. 2018. 10.7910/DVN/1WB9E4

[39] Uhl JH, Leyk S. Historical built-up property locations (BUPL)—gridded surfaces for the U.S. from 1810 to 2015. In: Harvard Dataverse [Internet]. 2020. 10.7910/DVN/SJ213V

[40] Uhl JH, Leyk S. Historical built-up property records (BUPR)—gridded surfaces for the U.S. from 1810 to 2015. In: Harvard Dataverse [Internet]. 2020. 10.7910/DVN/YSWMDR

[41] Uhl J H., Leyk S M. McShane C, E. Braswell A, S. Connor D, Balk D. Fine-grained, spatiotemporal datasets measuring 200 years of land development in the United States. Earth System Science Data. 2021;13: 119–153. doi: 10.5194/essd-13-119-2021 34970355 PMC8716019

[42] Uhl JH, Leyk S. Uncertainty surfaces accompanying the BUPR, BUPL, and BUA gridded surface series. In: Harvard Dataverse [Internet]. 2020. 10.7910/DVN/T8H5KF

[43] US Department of Homeland Security. FEMA Needs to Improve Management of Its Flood Mapping Programs, OIG-17-110. 2017. Available: https://www.oig.dhs.gov/sites/default/files/assets/2017/OIG-17-110-Sep17.pdf

[44] Office of Management and Budget. 2010 Standards for Delineating Metropolitan and Micropolitan Statistical Areas. Federal Register. 2010;75: 1–9. Available: www.whitehouse.gov/omb/fedreg/

[45] Balk D, Leyk S, Jones B, Montgomery MR, Clark A. Understanding urbanization: A study of census and satellite-derived urban classes in the United States, 1990–2010. PLOS ONE. 2018;13: e0208487. doi: 10.1371/journal.pone.0208487 30586443 PMC6306171

[46] Leyk S, Balk D, Jones B, Montgomery MR, Engin H. The heterogeneity and change in the urban structure of metropolitan areas in the United States, 1990–2010. Scientific data. 2019;6: 321. doi: 10.1038/s41597-019-0329-6 31844062 PMC6915769

[47] Landsea CW, Franklin JL. Atlantic hurricane database uncertainty and presentation of a new database format. Monthly Weather Review. 2013;141: 3576–3592. doi: 10.1175/MWR-D-12-00254.1

[48] USGS. Protected Areas Database of the United States (PAD-US). In: U.S. Geological Survey, Gap Analysis Project (GAP) [Internet]. 2018. Available: 10.5066/P955KPLE.

[49] USFWS. National Wetlands Inventory -Version 2—Surface Waters and Wetlands Inventory. In: U.S. Fish and Wildlife Service [Internet]. 2019. Available: https://www.fws.gov/wetlands/data/Data-Download.html

[50] Gormsen E. The impact of tourism on coastal areas. GeoJournal. 1997;42: 39–54.

[51] Glaeser EL, Tobio K. The rise of the sunbelt. National Bureau of Economic Research. 2007;74: 610–643. doi: 10.3386/w13071

[52] Baltagi B. Econometric analysis of panel data. 4th Edition. Chichester: John Wiley & Sons; 2008.

[53] Crossett KM, Culliton TJ, Wiley PC, Goodspeed TR. Population Trends Along the Coastal United States: 1980–2008. Coastal Trends Report Series. 2004.

[54] Lazarus ED, Limber PW, Goldstein EB, Dodd R, Armstrong SB. Building back bigger in hurricane strike zones. Nature Sustainability. 2018;1: 759–762. doi: 10.1038/s41893-018-0185-y

[55] Abbott C. Urbanizing the Sunbelt. OAH Magazine of History. 2003;18: 11–16.

[56] Gutmann MP, Field V. Katrina in historical context: Environment and migration in the U.S. Population and Environment. 2010;31: 3–19. doi: 10.1007/s11111-009-0088-y PMC2860332 20436951

[57] Fishman R. The American metropolis at century’s end: Past and future influences. Housing Policy Debate. 2000;11: 199–213. doi: 10.1080/10511482.2000.9521367

[58] Glaeser EL, Shapiro J. Is there a new urbanism? The growth of U.S. cities in the 1990s. National Bureau of Economic Research. 2001;w8357. doi: 10.3386/w8357

[59] Shepard CC, Agostini VN, Gilber B, Allen T, Stone J, Brooks W, et al. Assessing future risk: Quantifying the effects of sea level rise on storm surge risk for the southern shores of Long. Natural Hazards. 2012;60: 727–745. doi: 10.1007/s11069-011-0046-8

[60] Jagger T, Elsner JB, Niu X. A dynamic probability model of hurricane winds in coastal counties of the United States. Journal Applied Meteorology. 2001;40: 853–863.

[61] Malmstadt J, Scheitlin K, Elsner J. Florida hurricanes and damage costs. Southeastern Geographer. 2009;49: 108–131.

[62] Zhang K. Analysis of non-linear inundation from sea-level rise using LIDAR data: a case study for South Florida. Climatic Change. 2011;106: 537–565. doi: 10.1007/s10584-010-9987-2

[63] Huang X, Wang C, Lu J. Understanding the spatiotemporal development of human settlement in hurricane-prone areas on the US Atlantic and Gulf coasts using nighttime remote sensing. Natural Hazards and Earth System Sciences. 2019;19: 2141–2155. doi: 10.5194/nhess-19-2141-2019

[64] Demuth JL, Morss RE, Lazo JK, Trumbo C. The effects of past hurricane experiences on evacuation intentions through risk perception and efficacy beliefs: A mediation analysis. Weather, Climate, and Society. 2016;8: 327–344. doi: 10.1175/WCAS-D-15-0074.1

[65] Logan JR, Issar S, Xu Z. Trapped in Place? Segmented Resilience to Hurricanes in the Gulf Coast, 1970–2005. Demography. 2016;53: 1511–1534. doi: 10.1007/s13524-016-0496-4 27531504 PMC5050132

[66] Pais JF, Elliott JR. Places as recovery machines: Vulnerability and neighborhood change after major hurricanes. Social Forces. 2008;86: 1415–1453. doi: 10.1353/sof.0.0047

[67] Deyle RE, Chapin TS, Baker EJ. The proof of the planning Is in the platting: An evaluation of Florida’s Hurricane Exposure Mitigation Planning Mandate. Journal of the American Planning Association. 2008;74: 349–370. doi: 10.1080/01944360802229612

[68] Highfield WE, Peacock WG, van Zandt S. Mitigation Planning: Why Hazard Exposure, Structural Vulnerability, and Social Vulnerability Matter. Journal of Planning Education and Research. 2014;34: 287–300. doi: 10.1177/0739456X14531828

[69] FEMA. Hurricane Sandy FEMA After-Action Report. 2013. Available: https://www.fema.gov/media-library-data/20130726-1923-25045-7442/sandy_fema_aar.pdf

[70] Tomiczek T, Asce SM, Kennedy A, Asce M, Rogers S, Asce M. Collapse limit state fragilities of wood-framed residences from storm surge and waves during Hurricane Ike. Journal of Waterway, Port, Coastal and Ocean Engineering. 2014;140: 43–55. doi: 10.1061/(ASCE)WW.1943-5460.0000212

[71] Oppenheimer M, Glavovic BC, Hinkel J, van de Wal R, Magnan AK, Biesbroek R, et al. Sea Level Rise and Implications for Low-Lying Islands, Coasts and Communities. IPCC. In: Abe-Ouchi A, Gupta K, Pereira J, editors. IPCC. 2019.

[72] Sutton-Grier AE, Gittman RK, Arkema KK, Bennett RO, Benoit J, Blitch S, et al. Investing in natural and nature-based infrastructure: Building better along our coasts. Sustainability (Switzerland). 2018;10. doi: 10.3390/su10020523

[73] Haasnott M, Lawrence J, Magnan AK. Pathways to coastal restreat. Science. 2021;372: 1284–1287. doi: 10.1126/science.abh4283 34140381

[74] Gunawansa A, Kua HW. A comparison of climate change mitigation and adaptation strategies for the construction industries of three coastal territories. Sustainable Development. 2014;22: 52–62. doi: 10.1002/sd.527

