# Supplementary material for: Creeping disaster along the U.S. coastline: Understanding exposure to sea level rise and hurricanes through historical development
Source: PLoS One. 2022 Aug 3;17(8):e0269741. doi: 10.1371/journal.pone.0269741 (PMC9348716; doi:10.1371/journal.pone.0269741)
Supplement: S1 File — The S1 Table is an analysis of built up properties by a subset of counties. We used the ZTRAX raw data, 250 meter grid cells from HISDAC-US (BUPL; Uhl and Leyk 2020), and resampled grid cells used in the analysis for this paper to compare the number of structures across datasets. The S2 Table is the results from the built environment and cumulative storm hits regression analysis from 1900–2015 (with MSA-Year fixed effects). S1 Fig shows the Metro- and Micro- Statistical Areas (MSA) included in analysis. ZTRAX data is affected by issues of data incompleteness. We exclude states and MSAs from this study that has a significant percentage of grid cells (more than 50%) with low quality data (grid cell year built missingness < 5%). All data used in figure available through an open license for U.S. government datasets [30]. (DOCX) [file pone.0269741.s001.docx]

**Supporting Information**

***PLOS One***

**Title:** Creeping disaster along the U.S. coastline: Understanding exposure to sea level rise and hurricanes through historical development

**Contents:**

S1 Table

S2 Table

S1 Fig

**S1 Table.** **Analysis of built up properties by a subset of counties.** We used the ZTRAX raw data, 250-meter grid cells from HISDAC-US, and resampled grid cells used in the analysis for this paper.

|  |  |  | ***Raw Data*** | | ***250 meters*** | | ***50 meters*** | | |
| --- | --- | --- | --- | --- | --- | --- | --- | --- | --- |
| **County** | **State** | **FIPS** | **# Structures** | **Density (km^2^)** | **# Structures** | **Density (km^2^)** | **# Structures** | **Density (km^2^)** | **Percent of Raw Records** |
| Atlantic | NJ | 34001 | 133202 | 77 | 91384 | 58 | 91378 | 58 | 69 |
| Broward | FL | 12011 | 719312 | 649 | 456136 | 144 | 455856 | 144 | 63 |
| Chatham | GA | 13051 | 102606 | 63 | 79076 | 62 | 79069 | 62 | 77 |
| Escambia | FL | 12033 | 130104 | 57 | 110442 | 57 | 110397 | 57 | 85 |
| Fairfax | VA | 51059 | 351223 | 334 | 282023 | 271 | 282358 | 271 | 80 |
| Harris | TX | 48201 | 1278898 | 278 | 1078597 | 235 | 1078557 | 235 | 84 |
| King | WA | 53033 | 659800 | 110 | 534312 | 94 | 534046 | 94 | 81 |
| Los Angeles | CA | 06037 | 2295448 | 186 | 1872517 | 176 | 1872340 | 176 | 82 |
| Nassau | NY | 36059 | 405131 | 345 | 376686 | 461 | 376569 | 461 | 93 |
| New Hanover | NC | 37129 | 89961 | 106 | 74412 | 131 | 74292 | 131 | 83 |

**S2 Table. Built environment and cumulative storm hits regression analysis from 1900-2015** (with MSA-Year fixed effects).

| **Location** | **Coastal** | | **Coastal &**  **> 0 landfalls** | **Coastal** | | **Coastal &**  **> 0 landfalls** | **Coastal** | | **Coastal &**  **> 0 landfalls** |
| --- | --- | --- | --- | --- | --- | --- | --- | --- | --- |
|  | (1) | (2) | (3) | (4) | (5) | (6) | (7) | (8) | (9) |
|  | Share of  built-up land | Share of  built-up land | Share of  built-up land | Structure  density | Structure  density | Structure  density | Total  structures | Total  structures | Total  structures |
|  |  |  |  |  |  |  |  |  |  |
| Sea-level rise zone | 0.0775* (0.016) | 0.0507* (0.017) | 0.0428 (0.032) | 0.0572* (0.017) | 0.0375* (0.020) | 0.0102 (0.025) | -84.43* (28.039) | -62.86** (28.957) | 16.68 (33.663) |
|  |  |  |  |  |  |  |  |  |  |
| Cumulative storms | - | - | - | - | - | - | - | - | - |
|  |  |  |  |  |  |  |  |  |  |
| SLRZ x Cumulative  storms |  | 0.0307** (0.014) | 0.0327* (0.018) |  | 0.0226 (0.014) | 0.0295* (0.016) |  | -24.71 (29.136) | -44.98 (33.591) |
|  |  |  |  |  |  |  |  |  |  |
| Constant | 0.0932* (0.008) | 0.0932* (0.008) | 0.0842* (0.012) | 0.0378* (0.008) | 0.0378* (0.008) | 0.0363* (0.010) | 87.62* (14.019) | 87.62* (14.047) | 79.49* (21.140) |
|  |  |  |  |  |  |  |  |  |  |
| Observations | 4656 | 4656 | 2016 | 4656 | 4656 | 2016 | 4656 | 4656 | 2016 |
| *R*^2^ | 0.802 | 0.823 | 0.833 | 0.744 | 0.757 | 0.809 | 0.550 | 0.561 | 0.617 |
| Clustered SEs | MSA | MSA | MSA | MSA | MSA | MSA | MSA | MSA | MSA |
| Fixed effects | MSA x  Year | MSA x  Year | MSA x  Year | MSA x  Year | MSA x  Year | MSA x  Year | MSA x  Year | MSA x  Year | MSA x  Year |

Standard errors in parentheses

^*^ *p* < 0.10, ^**^ *p* < 0.05, ^*^ *p* < 0.01

**
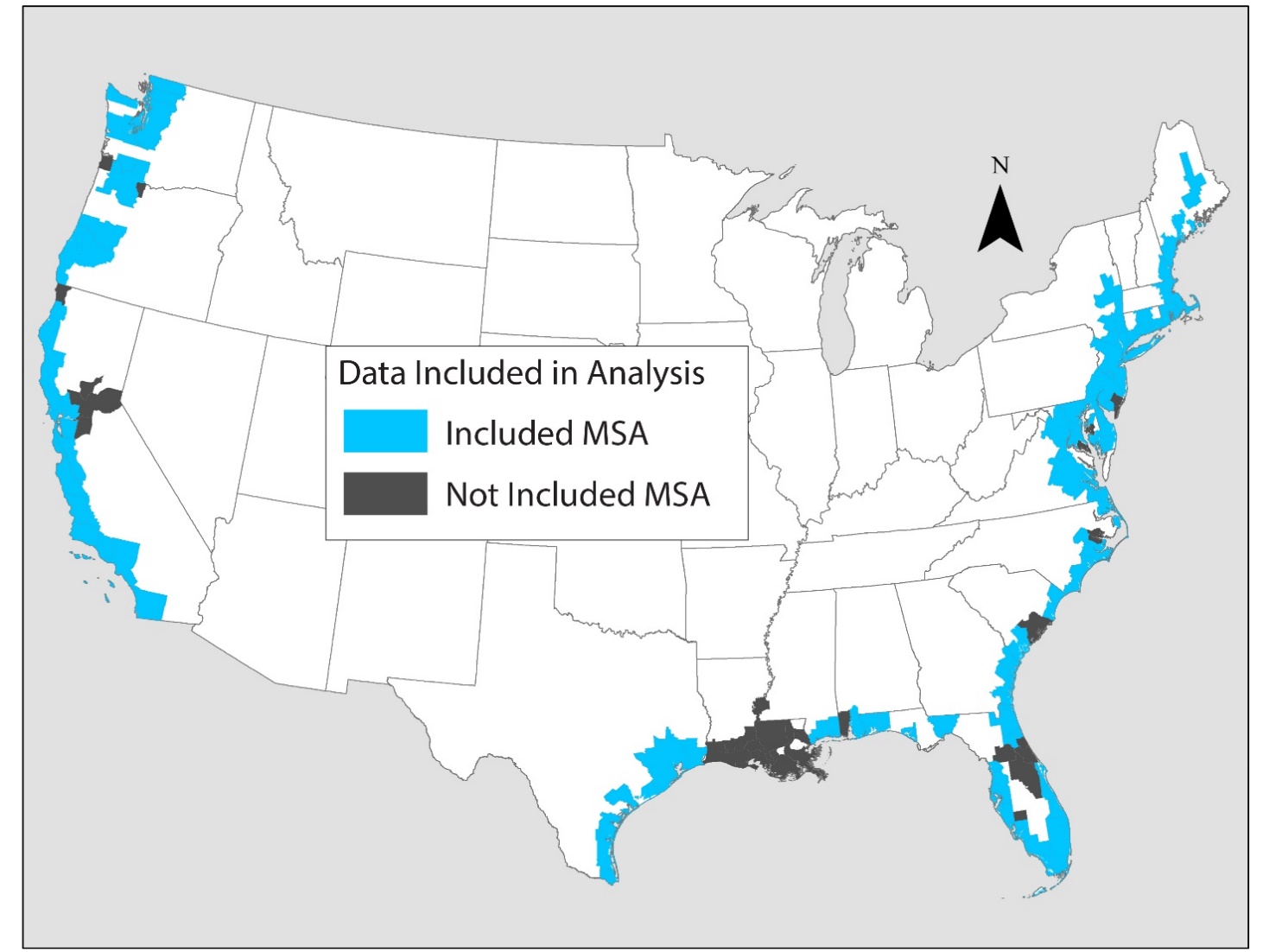
**

**S1 Fig. Metro- and Micro- Statistical Areas (MSA) included in analysis.** ZTRAX data is affected by issues of data incompleteness. We exclude states and MSAs from this study that has a significant percentage of grid cells (more than 50%) with low quality data (grid cell year built missingness < 5%).
